# Supplementary material for: In Vitro Antiprotozoal Activity of Schinus molle Extract, Partitions, and Fractions against Trypanosoma cruzi
Source: Plants (Basel). 2024 Aug 6;13(16):2177. doi: 10.3390/plants13162177 (PMC11359525; doi:10.3390/plants13162177)
Supplement: Supplementary file 1 [file plants-13-02177-s001.zip › plants-3112350-supplementary.pdf]

Figure S1

Fig S1. Chromatogram mass spectra of 20 different compounds obtained from the *S. molle* fractions. **Fig. S1A**. 1)1,4-Bis (trimethylsilyl) benzene; 2) Hexamethylcyclotrisiloxane; 3) Benzene, 4-ethyl-1,2-dimethyl; 4) 17-Octadecynoic acid; 5) 8-Chlorocapric acid. **Fig. S1B**. 6) Nonanoic acid; 7) Isopinocarveol; 8) Octanoic acid; 9) 9,12,15-Octadecatrienoic acid, 2-[(trimethylsilyl)oxy]-1-[[(trimethylsilyl)oxy]methyl]ethyl ester, (Z,Z,Z); 10) Desulphosinigrin, Formula: $C_{10}H_{17}NO_6S$, Mass: 279.077658, Rt: (27.195 min), % total: 19.5%. **Fig. S1C.** 11) 5,7-Dodecadiyn-1,12-diol; 12) Cyclopropanedodecanoic acid, 2-octyl-, methyl ester; 13) 13,16-Octadecadiynoic acid, methyl ester; 14) cis-5,8,11,14,17-Eicosapentaenoic acid; 15) Cholestan-3-ol, 2-methylene-, (3β,5α), Formula: $C_{28}H_{48}O$, Mass: 400.370516, Rt: (35.074 min), % total: 16.7%. **Fig.S1D.** 16) trans-Z-α-Bisabolene epoxide; 17) [1,1'-Bicyclopropyl]-2-octanoic acid, 2'-hexyl-, methyl ester; 18) 9,12-Octadecadienoyl chloride, (Z,Z); 19) Dodecanoic acid, 3-hydroxy; 20) 7-Methyl-Z-tetradecen-1-ol acetate. Formula, mass and retention time is cited in Table 3.

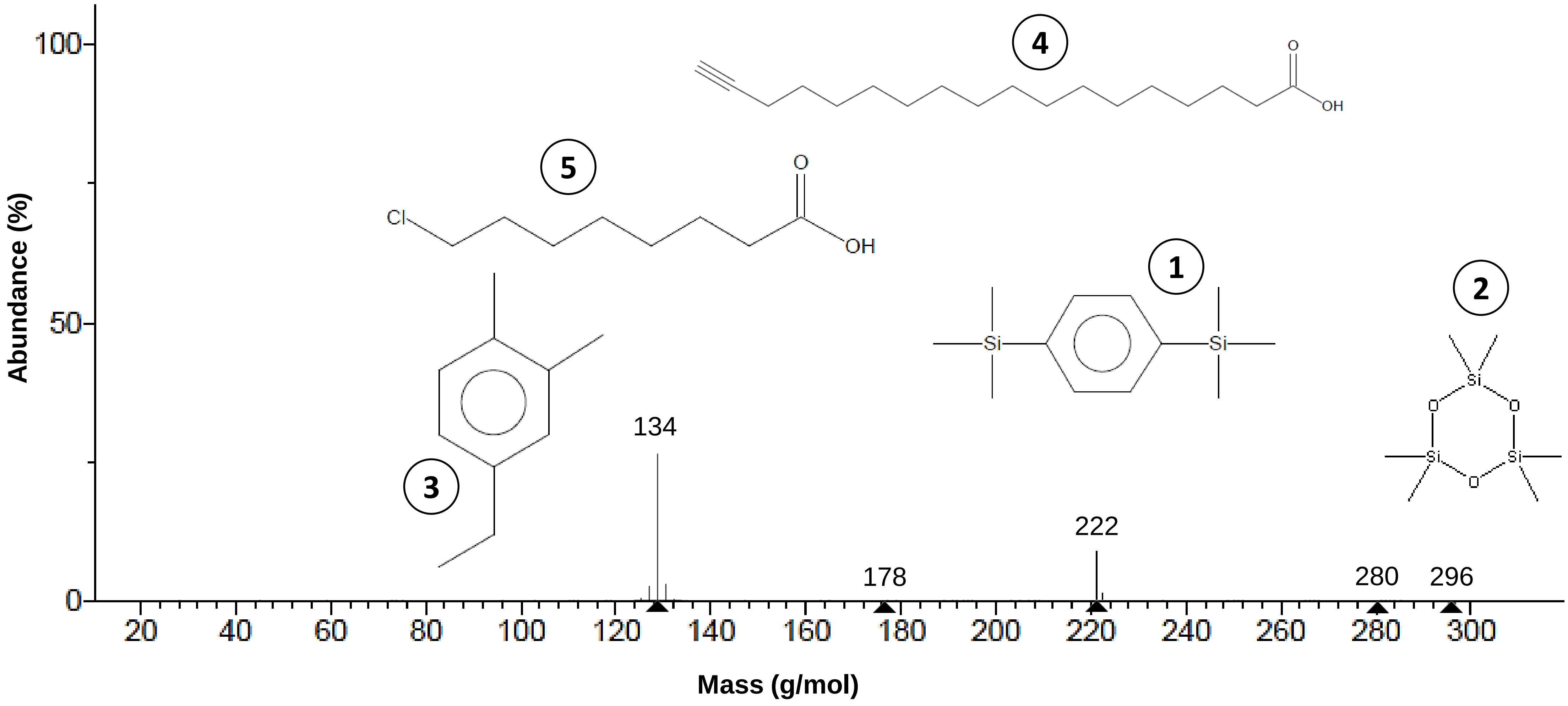

100
50
0
Abundance (%)
4
O
OH
5
O
Cl
OH
1
Si
Si
2
Si
O
O
Si
Si
O
3
134
222
178
280
296
20
40
60
80
100
120
140
160
180
200
220
240
260
280
300
Mass (g/mol)

Fig. S1A

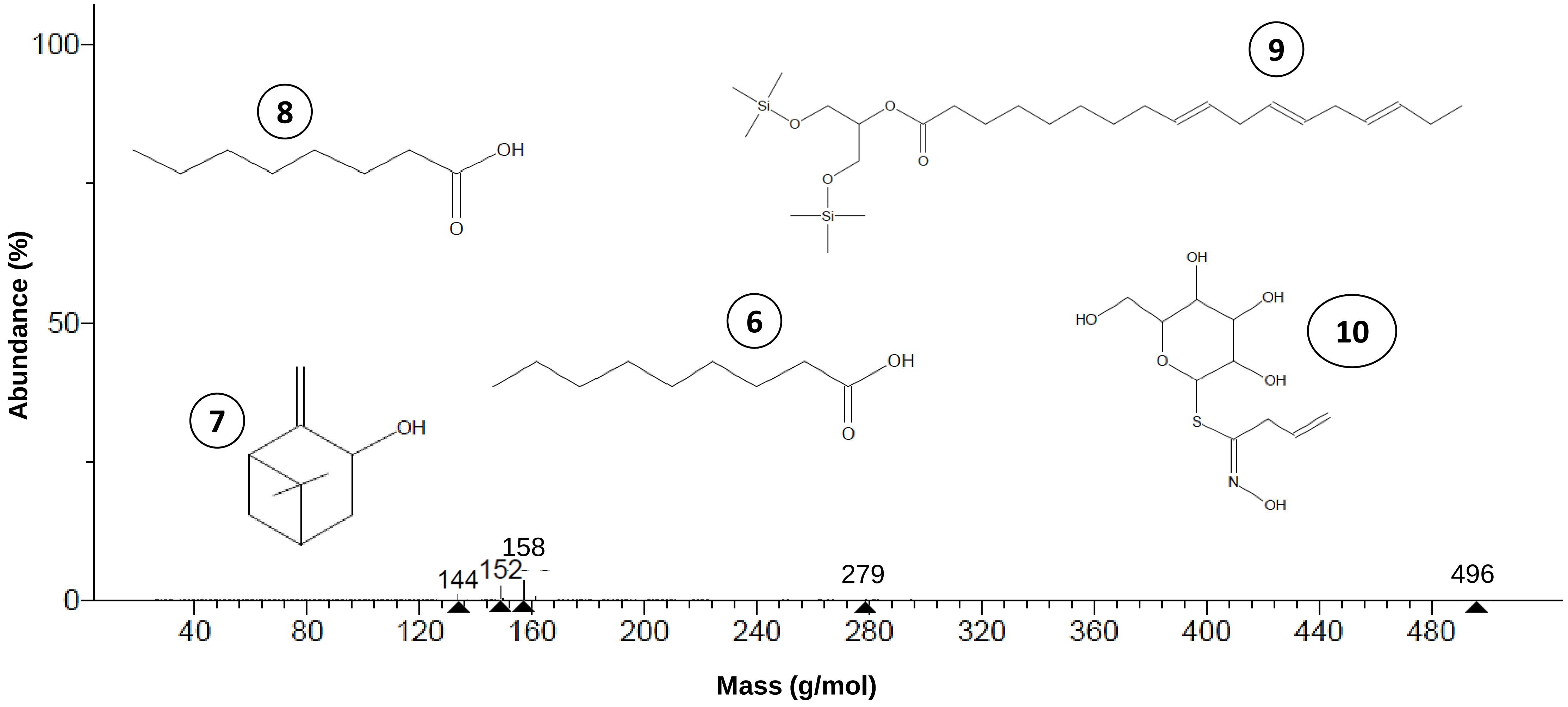

100
50
0
Abundance (%)
8
OH
O
9
Si
O
O
O
O
Si
6
OH
O
7
OH
HO
OH
OH
OH
O
OH
S
N
OH
10
158
144
152
279
496
40
80
120
160
200
240
280
320
360
400
440
480
Mass (g/mol)

Fig. S1B

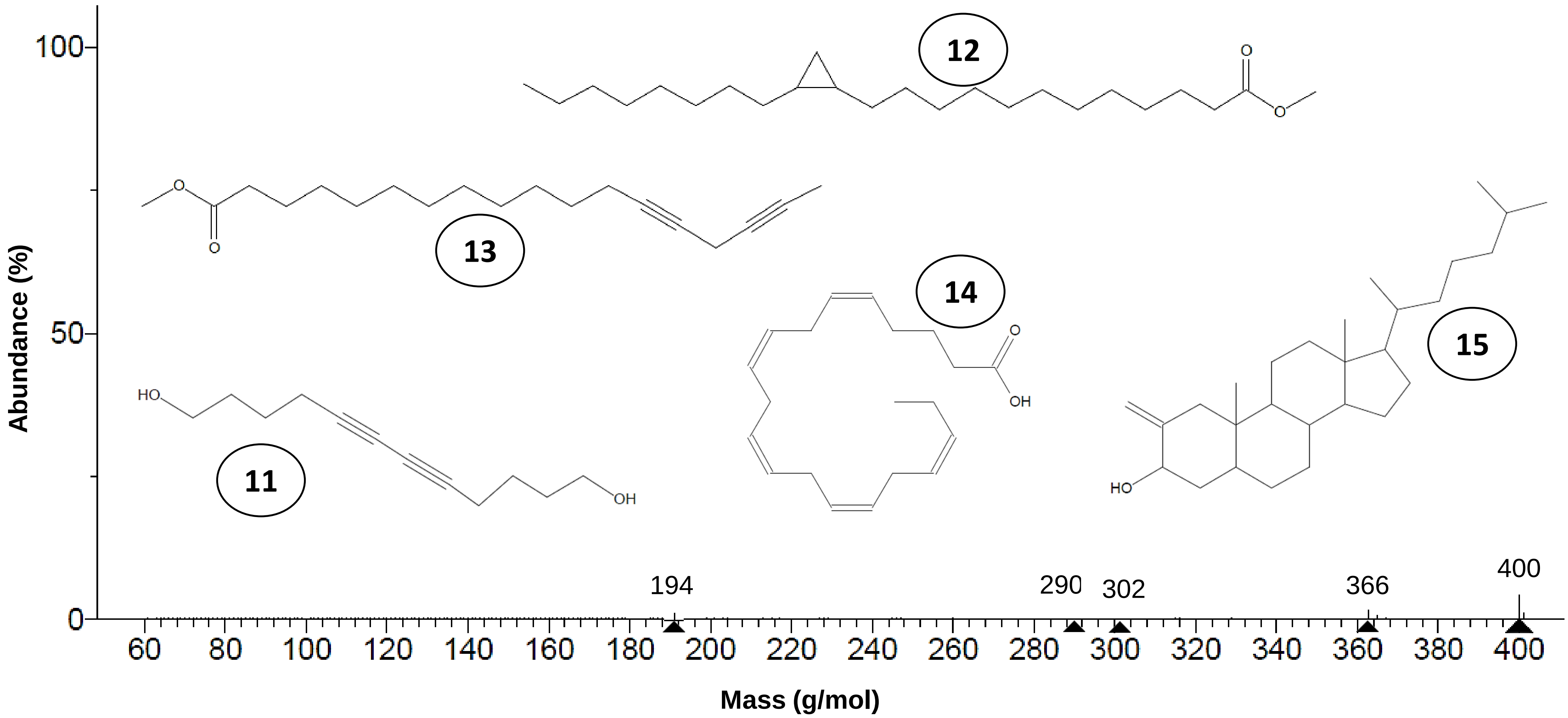

100
50
0
Abundance (%)
12
13
14
15
11
O
O
HO
OH
194
290
302
366
400
60
80
100
120
140
160
180
200
220
240
260
280
300
320
340
360
380
400
Mass (g/mol)

Fig. S1C

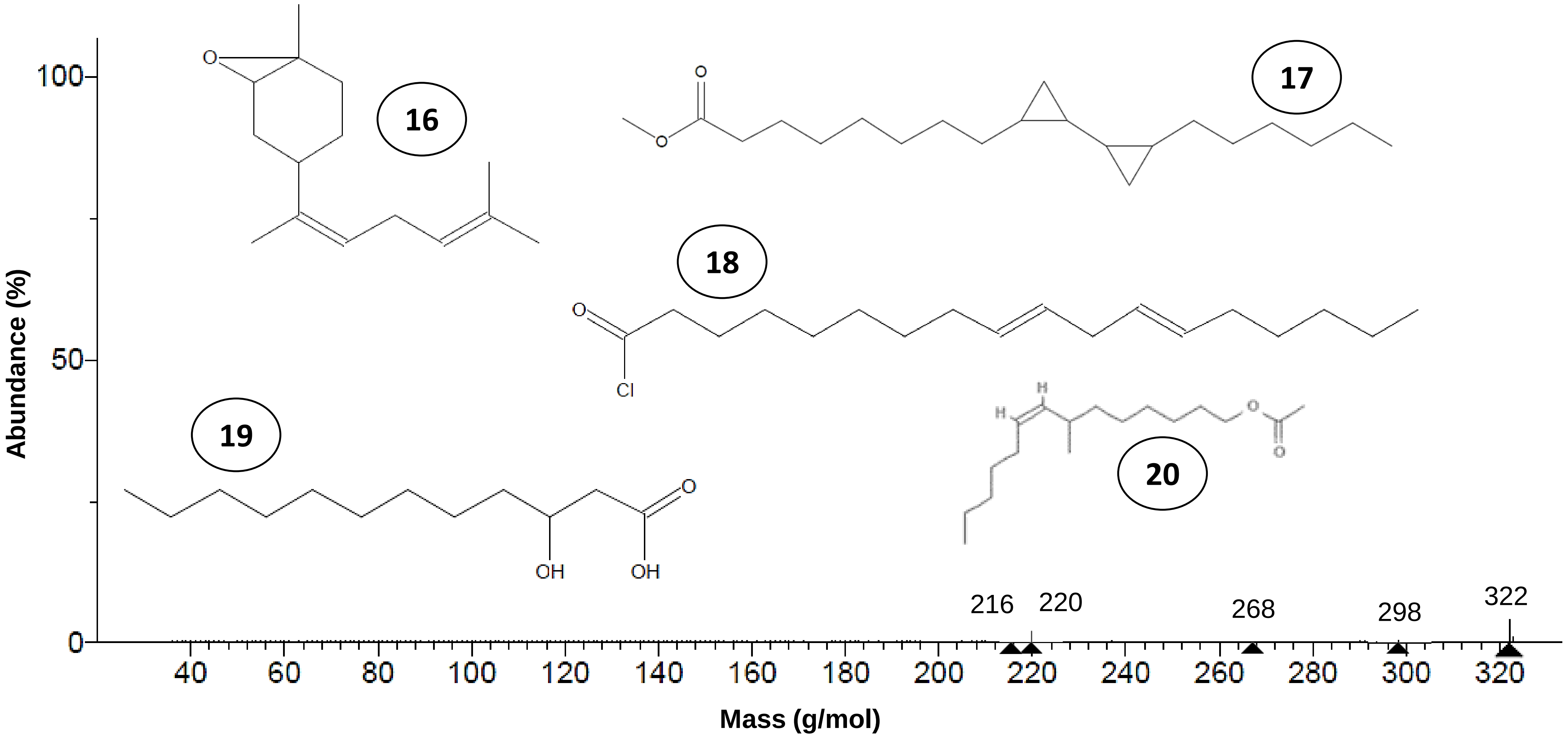
100
50
0
Abundance (%)
40
60
80
100
120
140
160
180
200
220
240
260
280
300
320
Mass (g/mol)
16
17
18
19
20
O
Cl
OH
216
220
268
298
322

Fig. S1D
